# The influence of a magnetic field on photon beam radiotherapy in a normal human TK6 lymphoblastoid cell line

**DOI:** 10.1186/s13014-019-1212-5

**Published:** 2019-01-17

**Authors:** B. Yudhistiara, F. Zwicker, K. J. Weber, P. E. Huber, A. Ruehle, S. Brons, P. Haering, J. Debus, S. H. Hauswald

**Affiliations:** 1 Department of Radiation Oncology, Heidelberg University Hospital, Im Neuenheimer Feld (INF) 400, 69120 Heidelberg, Germany; 2 Heidelberg Ion-Beam Therapy Center (HIT), Im Neuenheimer Feld 450, 69120 Heidelberg, Germany; 3 Clinical Cooperation Unit Molecular Radiation Oncology E055, German Cancer Research Center (DKFZ), Heidelberg, Germany; 4 Department of Radiation Physics E040, German Cancer Research Center (DKFZ), Heidelberg, Germany; 5 National Center for Radiation Research in Oncology (NCRO), Heidelberg Institute for Radiation Oncology (HIRO), Heidelberg, Germany; 6 Clinical Cooperation Unit E050, German Cancer Research Center (DKFZ), Heidelberg, Germany

**Keywords:** MRI guided radiotherapy, MR Linac, In-vitro experiment, Normal human cells, TK6 human lymphoblastoid cells

## Abstract

**Background:**

The implementation of magnetic resonance imaging (MRI) guided radiotherapy (RT) continues to increase. Very limited in-vitro data on the interaction of ionizing radiation and magnetic fields (MF) have been published. In these experiments we focused on the radiation response in a MF of the TK6 human lymphoblastoid cells which are known to be highly radiosensitive due to efficient radiation-induced apoptosis.

**Methods:**

Clonogenicity was determined 12–14 days after irradiation with 1–4 Gy 6 MV photons with or without a 1.0 Tesla MF. Furthermore, alterations in cell cycle distribution and rates of radiation induced apoptosis (FACS analysis of cells with sub-G1 DNA content) were analyzed.

**Results:**

Clonogenic survival showed an exponential dose-dependence, and the radiation sensitivity parameter (α = 1.57/Gy) was in accordance with earlier reports. Upon comparing the clonogenic survival between the two groups, identical results within error bars were obtained. The survival fractions at 2 Gy were 9% (without MF) and 8.5% (with MF), respectively.

**Conclusion:**

A 1.0 Tesla MF does not affect the clonogenicity of TK6 cells irradiated with 1–4 Gy 6MV photons. This supports the use of MRI guided RT, however ongoing research on the interaction of MF and radiotherapy is warranted.

## Introduction

Magnetic resonance image (MRI) guided radiation therapy (RT) is rapidly expanding owing to the superior soft tissue contrast compared with computer tomography (CT) [1]. Despite the tremendous effort in developing suitable technologies, concerns about possible alterations in biological responsiveness to a given therapeutic radiation dose in the presence of a magnetic field (MF) has found much less attention. While it is consented that current clinical MRI technologies can be safely applied [2], some observed physiological alterations [3] could become relevant when radiation damage is concomitantly being processed in a cell. However, such arguments are mere speculation at present. More obvious is the possibility that the released secondary electrons after photon energy absorption are subject to the Lorentz force in a MF. This would not only cause distortions of dose distribution at air/tissue interfaces [4] but could hypothetically also lead to clustering of DNA damage from such “backcircling” electrons [5, 6]. Only a few researchers have addressed this subject experimentally using different model systems, from baker’s yeast [7] to mouse mammary tumor cells [8] or Chinese hamster lung cells [9]. The common finding of these studies is a lack of statistically significantly increased radiation response due to static MF with field strength ranging from 0,14 to 2 Tesla. A similar observation was reported from our laboratory where the clonogenic survival of human tumor cells (WIDR colon adenocarcinoma and A549 lung carcinoma) was assessed after 6 MV photons (up to 8 Gy) with the absence or presence of a 1 Tesla static MF [10]. However, due to potential hazards from some unknown interaction phenomenon in the ionizing radiation-MF combination, it is reasonable to assume that the normal tissue cell response rather than the tumor cell response would be a more relevant question. Therefore, we assessed the radiation response of the TK6 human lymphoblastoid cells which are known to be highly radiosensitive due to efficient radiation-induced apoptosis; a mechanism frequently decreased or abrogated in tumor cells [11, 12].

## Methods

### Cell culture

The TK6 cell line with wild type p53 function was used in our experiments. TK6 cells (human lymphoblastoid cells from spleen) were originally provided by the Tumorbank of the German Cancer Research Center, DKFZ, Heidelberg, Germany. DNA cell line authentication was done by Eurofins Medigenomix Forensik GmbH, Ebersberg, Germany.

Identical to Schäfer’s work [11], the cells were cultured in suspension at 37 °C in a humidified atmosphere (6% CO_2_). The medium used was RPMI 1640 fortified with 10% heat-inactivated horse serum (Gibco) and 1% penicillin. The cell density was kept at 0.1 to 1.0 × 10^6^/ml by subculturing at regular intervals.

### Magnetic field

**Fig. 1:**
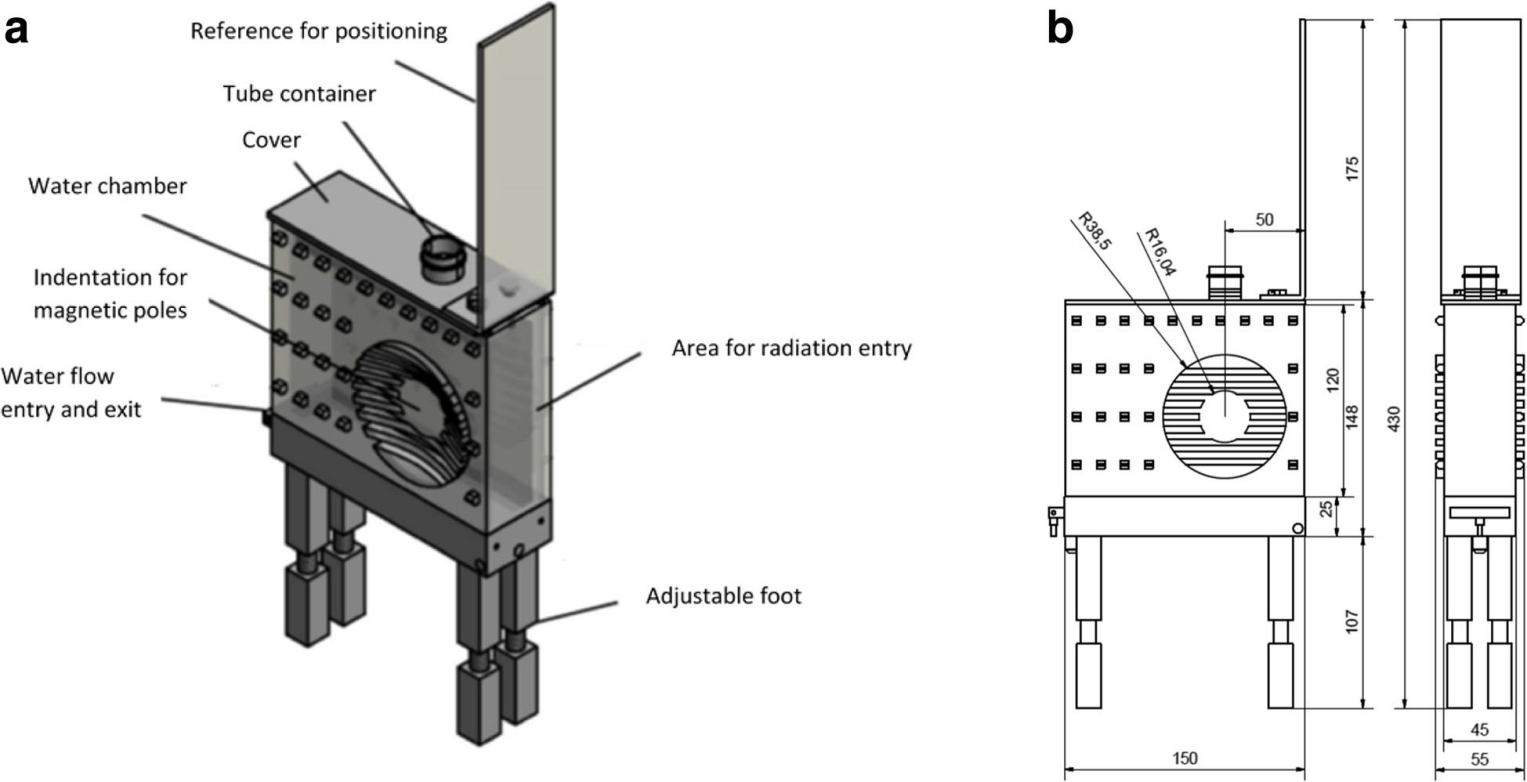
**(a)** A blueprint of the apparatus that was used in our experiments along with its dimensions measured in mm **(b)**. © Armin Runz, DKFZ

**Fig. 2:**
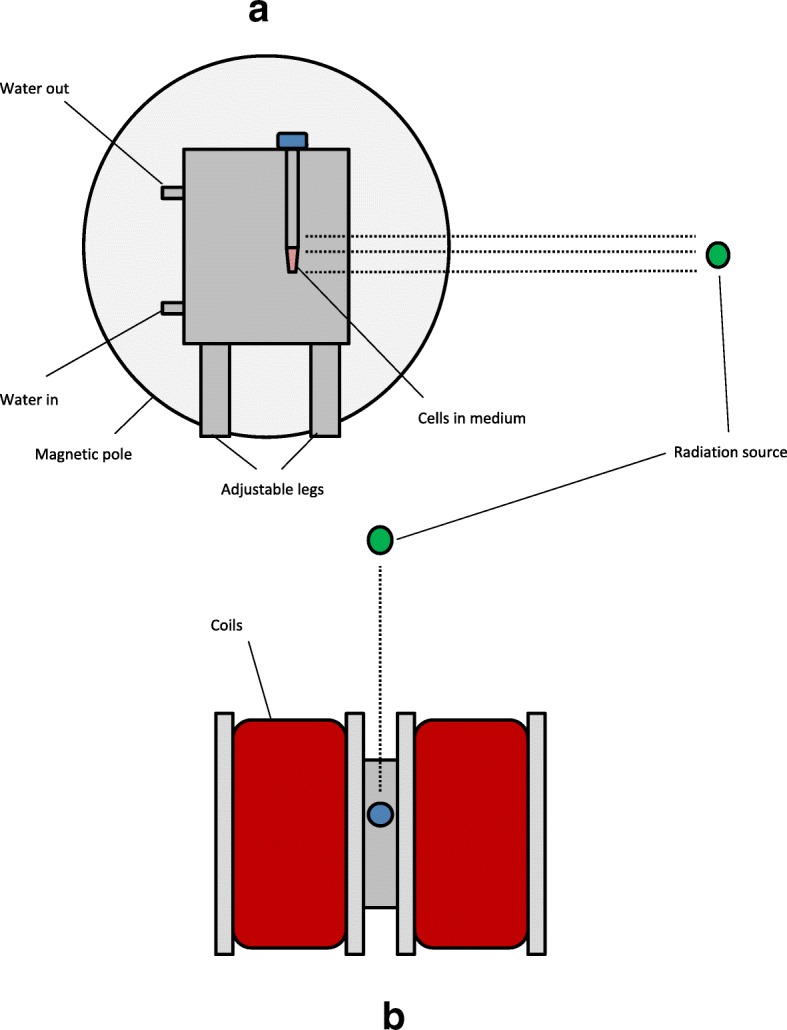
Diagrams showing the experimental set-up (not drawn to scale) in lateral **(a)** and aerial **(b)** views

The MF was generated using a pair of magnetic coils with an adjustable distance between the two poles, where MF of up to 1.5 Tesla can be generated upon allowing an electric current to flow to the coils. The amount of current needed to generate the desired MF strength was previously determined using a probe placed between the coils, from which the MF strength can be read off. A special container made of VeroClear RGD810 (Fig. 1) was then placed between the poles, creating a 3.5 cm space between the poles, in which the test tube containing the cells were to be placed. To help dissipate the heat generated from the electric current, creating a water-cooling system ensured a constant water flow into and out of the phantom, such that no cell deaths occurred due to overheating. Using the in-room laser positioning system, the cells were placed in the isocenter of the linear accelerator. Following the setup in Ziles’ work [10], the irradiation field was 10 cm in length and 3.3 cm in width, which covers the whole tube containing the cells. The overall experiment setup with its relevant parameters can be seen in Figs. 1 and 2.

### Radiation and Clonogenic survival

Photon beams were generated using a linear accelerator model Siemens Artiste 2 (6 MV) at the German Cancer Research Center (DKFZ). The clonogenic survival of the TK6 cells was determined by plotting their survival curves with and without MF. Raw data of vitality are obtained as plating efficiencies, and these values are determined in at least three independent experiments. Since our cells are not adherent in nature, the microtiter assay using the 96 well plates was used. Cultured cells were centrifuged, their supernatant discarded and then resuspended with 10 ml fresh medium.

A concentration of 3.0 × 10^5^ cells ml^− 1^ was then used for each test tube. The test tubes were chilled on ice in an ice box before and after irradiation to slow down any metabolic processes. After irradiation the cells were to be plated in the 96 wells such that each well has a specific number of cells. Table 1 summarizes the number of cells used in our experiments, according to their radiation types and respective doses. This number was determined through several pilot tests, such that the number of wells without cell colony after irradiation lies between 40 and 50 (see eq. (1)).

**Table 1:** The number of cells plated within a single well after irradiation with respect to the type of radiation used in the experiments

Type of radiation	Dose [Gy]	Number of cells per well
Photon beams	0	1
	1	2
	2	20
	3	50
	4	100

After 14 days the number of wells which have changed in color from red to yellow was counted for further calculations.

The plating efficiency (PE) is calculated as such:

1 $$ PE=\frac{1}{N}\bullet \ln \left(\frac{96}{n}\right) $$

Where N = the number of cells plated in a well, n = the number of wells without cell growth. Schäfer’s work has shown that n should lie between 40 and 50 to obtain stable results [11].

With the PE value, the survival fraction (SF) is then expressed as:

2 $$ SF=\frac{PE(treatment)}{PE(control)} $$

Where PE (control) is the plating efficiency obtained at 0 Gy (with and without MF) and PE (treatment) is the one obtained after the cells are irradiated. From three independent experiments the mean SF along with its standard deviation for each dose was also calculated (corrected to three decimal places or at least three significant figures). A 2-sample t-test was then performed comparing the two mean values between the control group (without MF) and treatment group (with MF). The difference between the two mean values is statistically significant when the *p*-value is < 0.05. A regression analysis was then performed following the linear quadratic model (LQ-model), which then defines the SF as an exponential function of the dose (D):

3 $$ SF={e}^{\left(-\alpha D-\beta {D}^2\right)} $$

α and β are coefficients that can then be determined using the regression analysis.

### Fluorescence-activated cell sorting (FACS)

FACS was used to determine the shift in the cell cycle population as well as the treatment specific apoptosis. Past works have demonstrated the reliability of this method to determine the rate of radiation-induced apoptosis [13]. Irradiated cells were cultured in a new flask containing a new medium for 8, 10, 12, 14, 24 and 48 h respectively, after which the cells were fixated using 1 ml of 80% alcohol and stored in the refrigerator at 10 °C. For analysis, the alcohol solution was discarded and the cells centrifuged. Afterwards they were rinsed with 2 ml of phosphate-buffered saline (PBS) twice (with centrifugation and supernatant discard after each rinse). To stain them, we added 900 μl of PBS and 100 μl of propidium iodide. The tubes were kept in the refrigerator at 10 °C for 12 h to maximize staining. The changes in the percentage of cells in each phase could thereby be tracked. To measure the rate of apoptosis after treatment, the percentage of sub-G1 population of the cells was determined. Afterwards, the treatment-specific apoptosis (TSA) was calculated with the aid of the following formula:

4 $$ TSA=\frac{f_x-{f}_0}{1-{f}_0} $$

Where f_x_ is the total sub-G1-phase fraction after treatment and f_0_ is the respective value of the untreated control (non-irradiated cells) from the same experiment. We performed four repeats of this experiment independently and calculated the mean TSA. Similar to the survival assay, a two-sample unpooled t-test was also performed to determine whether the differences in the TSA between the two groups (with and without MF) are statistically significant. Here the difference between the two mean values is statistically significant when the *p*-value is < 0.05 as well.

### Two-factor analysis of variance (two-way ANOVA)

We decided to include two-way ANOVA as an additional statistical tool, which compares all variances in the control group to those in the treatment group. This analysis was generated using SPSS 23 by IBM.

## Results

### Clonogenic assay

**Fig. 3:**
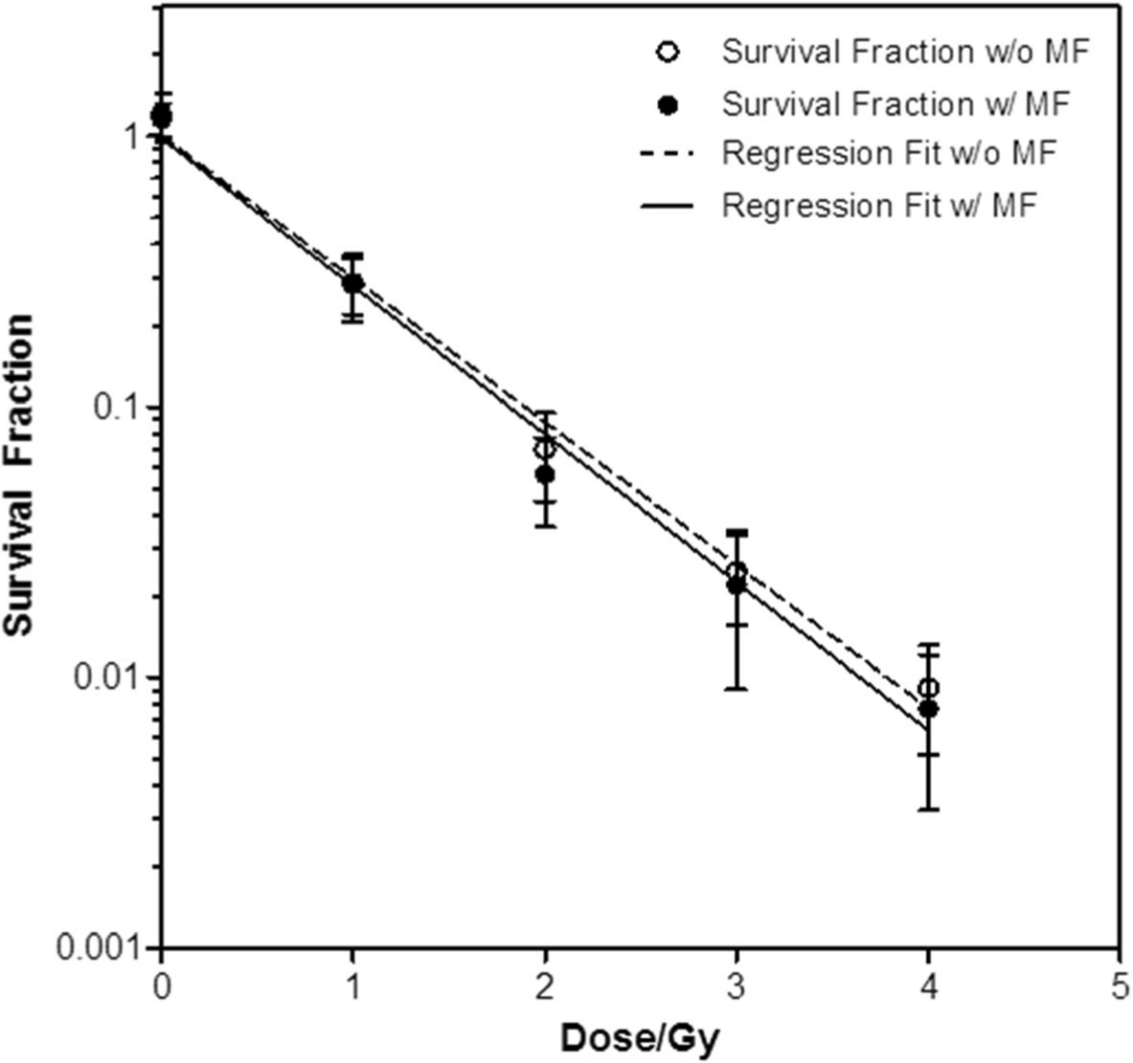
The survival curve generated after regression analysis following the LQ model. Note the proximity of the two curves (with or without magnetic field (MF)) and the overlapping error bars between the mean survival fractions

Table 2 shows the mean SF of TK6 human lymphoblastoid cells after irradiation and 14 days incubation. Each mean SF was calculated from 3 independent experiments using the formulae previously described. The corresponding t-test and *p*-values are listed. The survival curves are shown in Fig. 3.

**Table 2:** The mean survival fractions (SF) of 3 independent experiments with and without MF with the respective t-test and *p* values

Dose [Gy]	Mean SF (without MF)	σ	Mean SF (with MF)	σ	t-test	*p*-value
0	1.191	0.238	1.155	0.162	0.217	0.840
1.0	0.286	0.0800	0.286	0.0679	0.00	1.00
2.0	0.0698	0.0251	0.0563	0.0202	0.726	0.510
3.0	0.0246	0.00904	0.0220	0.0130	0.284	0.792
4.0	0.00915	0.00398	0.00767	0.00444	0.430	0.690

### Cell cycle analysis

**Fig. 4:**
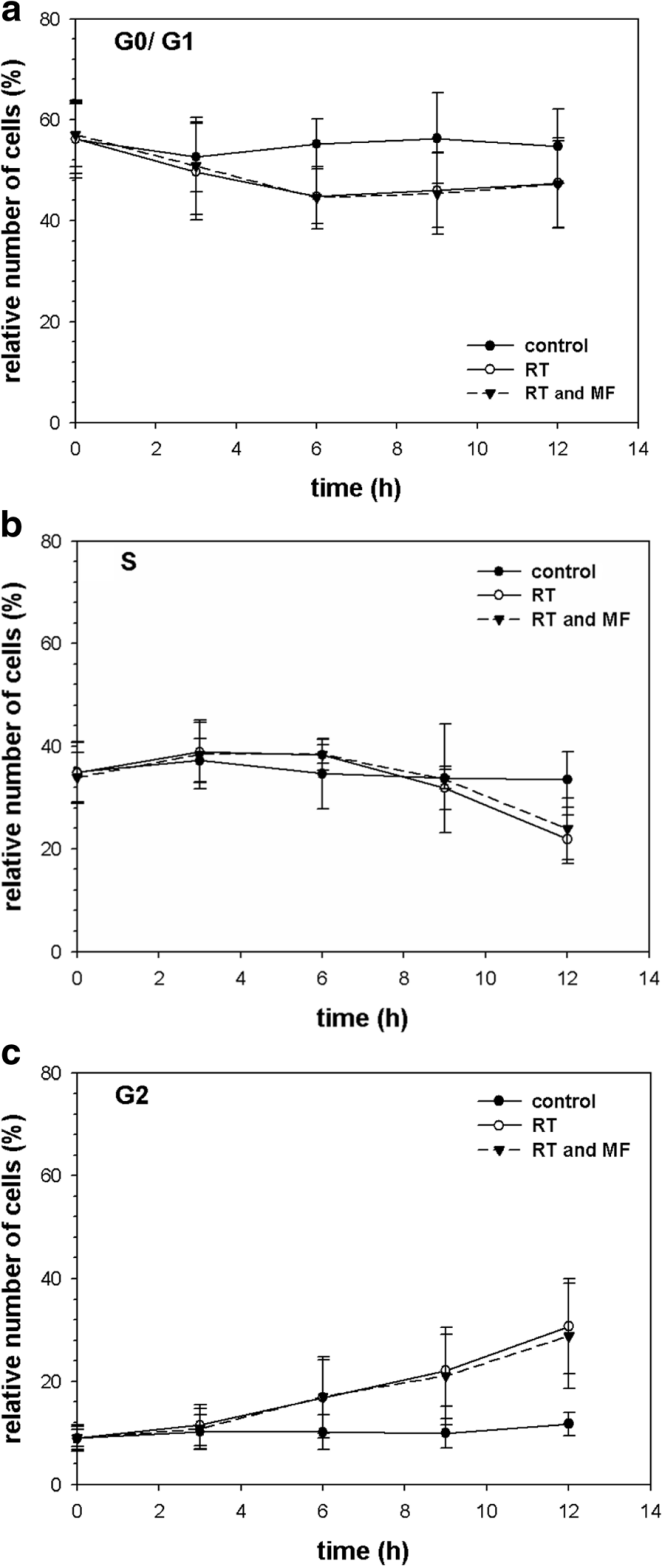
Cell-cycle analysis with the aid of FACS:(**a**) G1/G0 (**b**) S and (**c**) G2 show the relative number of cells after irradiation with 4 Gy photons (RT) with or without magnetic field (MF). Un-irradiated controls were performed in each experiment. Error bars showed the standard deviation of four independent experiments

Figure 4 shows the results of four identical, independent FACS-analyses of the cell cycle progression (G1/G0, S and G2) after photon irradiation of 4 Gy. After 12 h the irradiated cells showed a significantly increased number of cells in the G2 phase compared to the controls (*p*-value < 0.05) regardless of the presence of a MF. Furthermore, there was no significant difference in the progression of the cell cycle between the cells which were irradiated with a MF and those without a MF (*p*-value > 0.05 for all time intervals and measurement points). Hence, the MF has no influence on the cell cycle progression after photon irradiation in TK6 cells.

Note the falling G0/G1 population with the simultaneous increase in G2 population over time in all four experiments. Also, the solid and dotted curves are almost identical for their respective cell cycle phase in each experiment.

### Treatment-specific apoptosis

**Fig. 5:**
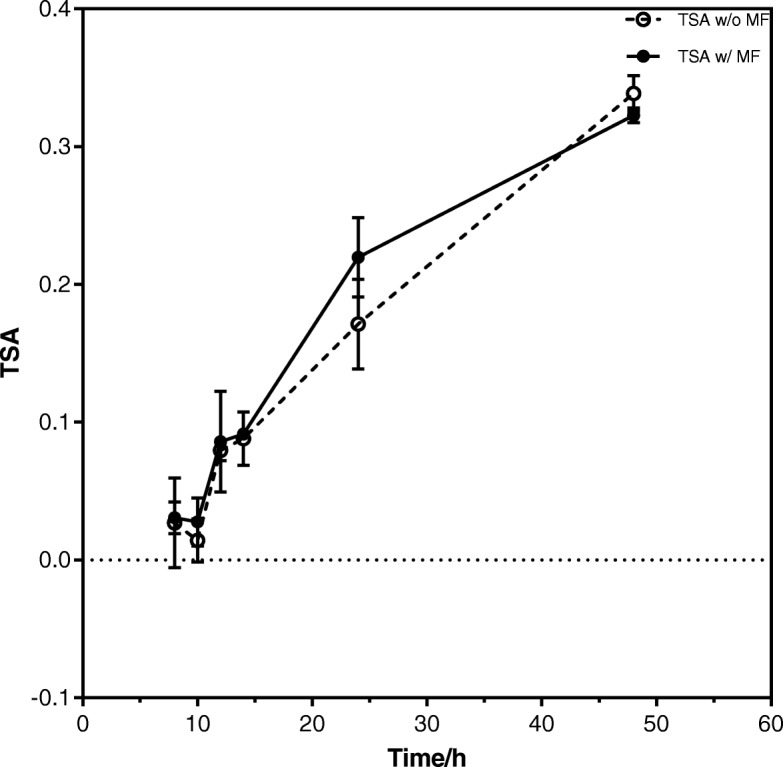
Treatment-specific apoptosis (TSA) 8, 10, 12, 14, 24 and 48 h after irradiation of TK6 cell lines with 4 Gy photons both in the presence and absence of a magnetic field (MF) of 1 Tesla

Figure 5 shows the calculated treatment-specific apoptosis (TSA, eq. (4)) plotted against the incubation time after irradiation. As expected, the TSA shows an upward trend as the incubation time increases. The plotted values are shown in Table 3 (corrected to two decimal places or three significant figures).

**Table 3:** The calculated mean TSA of both groups with their respective t-test and p values for each time period. Each mean value was derived from 3 independent experiments

Time [h]	Mean TSA (without MF)	σ	Mean TSA (with MF)	σ	t-test value	*p*-value
8	0.0269	0.0326	0.0306	0.0115	0.182	0.869
10	0.0141	0.0157	0.0276	0.0174	1.004	0.373
12	0.0795	0.00738	0.0858	0.0366	0.292	0.796
14	0.0880	0.0194	0.0914	0.00279	0.303	0.790
24	0.171	0.0327	0.220	0.0288	1.58	0.191
48	0.339	0.0130	0.323	0.00534	1.59	0.222

### Two-way ANOVA

The ANOVA tables of each measured parameter are shown below (Tables 4 and 5). N = the number of statistical cases. df = degree of freedom. F = F-value used in two-way ANOVA. Sig. = Significance/p-value.

**Table 4:** Survival Fraction

Tests of Between-Subjects Effects					
Dependent Variable: Survival Fraction					
Source	Type III Sum of Squares	df	Mean Square	F	Sig.
Corrected Model	5.875^a^	9	.653	68.657	.000
Intercept	2.896	1	2.896	304.610	.000
Magnetic Field	.001	1	.001	.087	.770
Dose	5.873	4	1.468	154.421	.000
Magnetic Field * Dose	.001	4	.000	.036	.997
Error	.190	20	.010		
Total	8.961	30			
Corrected Total	6.065	29			

a. R Squared = .969 (Adjusted R Squared = .955)

**Table 5:** Treatment-specific Apoptosis

Tests of Between-Subjects Effects					
Dependent Variable: TSA					
Source	Type III Sum of Squares	df	Mean Square	F	Sig.
Corrected Model	.427^a^	11	.039	69.808	.000
Intercept	.560	1	.560	1007.354	.000
Magnetic Field	.001	1	.001	1.601	.218
Time	.422	5	.084	152.036	.000
Magnetic Field * Time	.003	5	.001	1.223	.329
Error	.013	24	.001		
Total	1.000	36			
Corrected Total	.440	35			

a. R Squared = .970 (Adjusted R Squared = .956)

## Discussion

The implementation of MRI-guided RT has been increasing in recent years with new MR-Linacs being installed in various centers around the world. However, only few data on the interaction of photon beam radiation and MF have been published. In this developing new field of RT, it is important to analyze potential interaction phenomena in the ionizing radiation-MF combination on tumor cell lines and normal tissue cell lines as well. Therefore, the radiation response of TK6 human lymphoblastoid cells which are known to be highly radiosensitive was analyzed as an indicator for normal tissue interactions. Since most MRI used in clinical settings utilize a MF of 0.5 to 1.5 T, our experiments were carried out in a MF of 1 T.

Raw data of vitality were obtained as plating efficiencies, and these values were determined in at least three independent experiments. Interexperimental variability of data originates from both stochastic errors (i.e. precision of pipetting) and from systematic changes of biological factors. The latter is a common change of inherent plating efficiency of a particular cell preparation – at a given day and subcultivation history – which needs to be accounted for by intraexperimental normalization before repeat experiments can be compared. Accordingly, two approaches can be distinguished: (i) all data (plating efficiencies of a particular experiment) are normalized to the respective control value yielding the surviving fractions in the simplest manner. This procedure, however, implies that the control value would have been determined without error, which is not true. (ii) The mathematical function used to describe SF as a function of dose (the LQ model) is fitted to all plating efficiencies of a particular experiment (including control). This yields a calculated (“the best”) value at dose zero which is then taken to normalize the plating efficiencies for each independent experiment. Subsequently, mean values (and standard deviations) from the repeat measurements were calculated and the fit procedure (i.e. with LQ model) was applied again.

In our experiments the regression of the SF for the photon beams produced a value of 1.57 for α, which is an indicator of radiosensitivity of the cell type. The linearity of the curve also confirms the radiosensitivity of the TK6 cell line used. This corresponds to Schäfer’s work [11] and therefore supports the reliability of the chosen experimental setup.

Given the observation of a lacking influence with regard to the MF, the respective statistical tests must be considered. For the clonogenic assay a 2-sample t-test was performed comparing the mean SF between the control and experimental groups. It is then assumed that the two groups that are being tested are independent of each other and that they are normally distributed. The unpooled t-test was selected since it is also assumed that the variances are not equal. Since *p* > 0.05 in all dose groups in all 3 different radiation types, it cannot be concluded that there is a statistically significant difference between the two mean SF. Similar results were observed when the TSA of both groups were compared against each other. A *p*-value > 0.05 for all points in the TSA graph also means that no conclusion about a statistically significant difference between the mean TSA of both groups can be reached. In other words, it cannot be concluded that there is a biologically relevant effect on TK6 cells after being irradiated in a MF.

In addition the two-way ANOVA test confirms our hypothesis. The two independent variables are the presence of a MF and irradiation dose. The significant values listed in all tables in the row “Magnetic_Field” are more than 0.05, which indicates that at 5% significance level, the presence of a MF does not have any statistically significant effect on the dependent variable, be it SF or TSA. Moreover, in the significant values in the row “Magnetic_Field * Dose” are all close to 1.00, which means that there is no interaction between those two independent variables. Hence through these statistical tests we can conclude that there are neither negative nor positive effects observed when TK6 cells are irradiated in a MF of 1 T.

In the cell cycle experiments we observed the well-known G2-Arrest after photon irradiation [14]. The addition of a MF had no significant influence on the cell cycle progression after photon irradiation in TK6 cells in our setting.

In another experiment from our laboratory on the clonogenic survival of human tumor cells (WIDR colon adenocarcinoma and A549 lung carcinoma cell lines) treated with photon beam RT in a 1 Tesla MF, comparable results to this report were found [10].

In summary, the present phenomenological experiment with normal human TK6 lymphoblastoid cell lines as a highly radiosensitive in-vitro system does not indicate any interaction of a static 1 Tesla MF on 1–4 Gy photon beam RT. Further research on potential interactions of MF and photon as well as particle beam treatments is warranted.
